# Estimation of the impact of three different bioinformatic pipelines on sheep nemabiome analysis

**DOI:** 10.1186/s13071-022-05399-0

**Published:** 2022-08-11

**Authors:** Paulius Baltrušis, Peter Halvarsson, Johan Höglund

**Affiliations:** Section for Parasitology, Department of Biomedical Sciences and Veterinary Public Health, Swedish University of Agricultural Sciences, Uppsala, Sweden

**Keywords:** Nemabiome, PacBio, Ivermectin, Albendazole, Monepantel, Anthelmintic resistance, Bioinformatic pipeline, NGS

## Abstract

**Background:**

Next-generation sequencing (NGS) has provided an alternative strategy to study the composition of nematode communities with increased resolution and sensitivity. However, the handling and processing of gigabytes worth of amplicon sequence data produced by an NGS platform is still a major hurdle, limiting the use and adoption of faster and more convenient analysis software.

**Methods:**

In total 32 paired, fecal samples from Swedish sheep flocks were cultured and the larvae subsequently harvested subjected to internal transcribed spacer 2 (ITS2) amplicon sequencing using the PacBio platform. Samples were analyzed with three different bioinformatic pipelines, i.e. the DADA2, Mothur and SCATA pipelines, to determine species composition and richness.

**Results:**

For the the major species tested in this study (*Haemonchus contortus, Teladorsagia circumcinta* and *Trichostrongylus colubriformis*) neither relative abundances nor species diversity differed significantly between the three pipelines, effectively showing that all three analysis pipelines, although different in their approaches, yield nearly identical outcomes. In addition, the samples analyzed here had especially high frequencies of *H. contortus* (90–95% across the three pipelines) both before and after sample treatment, followed by *T. circumcinta* (3.5–4%). This shows that *H. contortus* is the parasite of primary importance in contemporary Swedish sheep farms struggling with anthelmintic resistance. Finally, although on average a significant reduction in egg counts was achieved post-treatment, no significant shifts in major species relative frequencies occurred, indicating highly rigid community structures at sheep farms where anthelmintic resistance has been reported.

**Conclusions:**

The findings presented here further contribute to the development and application of NGS technology to study nemabiome compositions in sheep, in addition to expanding our understanding about the most recent changes in parasite species abundances from Swedish sheep farms struggling with anthelmintic resistance.

**Graphical Abstract:**

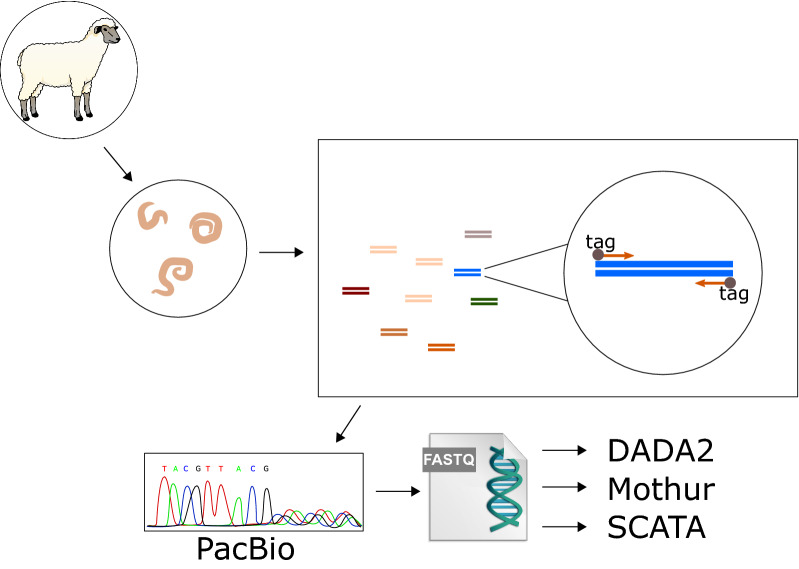

**Supplementary Information:**

The online version contains supplementary material available at 10.1186/s13071-022-05399-0.

## Background

Infections with helminth parasites are a commonplace occurrence on farms worldwide. In general, illnesses associated with gastrointestinal nematode species can range anywhere from mild diarrhea or lethargy to anemia and even death of the infected animal. Since therapeutic compounds offer the most reliable removal of parasites from their hosts, costs of treatment together with loss of productivity in livestock (e.g. slower weight gain) both result in significant economic losses in the animal production sector [1]. Furthermore, resistance development in the parasitic nematode communities have the potential to not only limit the drugs available to treat infected herds [2–5] but could also lead to the selection of species that are more pathogenic and more prone to developing resistance [6]. As a consequence, the balance in naturally occurring, complex communities could be shifted wherein the more pathogenic species and/or the species more prone to resistance development become more likely to be spread across farms.

In Swedish sheep, although known for some time, the major species of gastrointestinal nematodes have recently been confirmed to be *Haemonchus contortus*, *Teladorsagia circumcinta* and *Trichostrongylus vitrinus* [6], all of which belong to the same order—Strongylida. The dominant presence of these species appears to be somewhat similar in other countries with a temperate climate, such as Canada [7] and the UK [8], despite there being differences in other factors, such as geographic locations, pasture management techniques and treatment regimes. Nevertheless, comparatively little is known about the interactions of these different species, how such interactions affect the host as well as the influence and the significance of the more rarely encountered species.

The current “gold standard” methodology to estimate the surviving fraction of nematode parasites after treatment and community compositions in flocks typically relies on counting the remaining nematode eggs present in the feces of the infected animals and comparing these counts with those obtained prior to the applied treatment, with subsequent culturing of the infective third-stage larvae (L3) for species identification. The fundamental issues with performing fecal egg counts (FEC) stem from the fact that the obtained estimates are only approximate due to the general lack of sensitivity, specificity and repeatability of such assays, whereas the culturing of L3 involves an incubation period of at least 7–14 days as well the need for experts proficient in microscopically distinguishing fine morphological differences between the larvae. On the other hand, while multiplex [9], tandem [10] and droplet digital PCR [11] approaches can be successfully employed to evaluate the presence as well as the relative abundance of gastrointestinal nematode (GIN) species in flock samples, the throughput of such assays is generally a limiting factor as the detection and estimation of the abundance of parasites can only occur for a relatively small number of species for which the assay is optimized a priori and each sample has to be analyzed individually.

The more modern approach to analyzing the compositions of nematode communities is through the use of next-generation sequencing (NGS) analysis of multiplexed libraries containing amplified nematode internal transcribed spacer 2 (ITS2) DNA sequence fragments. Such approaches have already been successfully applied to study the nematode community compositions in beef cattle [12], sheep [6, 8], bison [13] and horses [14]. Although relatively novel in the field of veterinary parasitology, the application of this strategy for analyzing nematode communities is not only likely to yield more precise and sensitive estimations of parasite community structure and of the impact of external factors (such as treatment) on the changes in community compositions, but also allows for less laborious and highly robust large-scale farm screenings [7, 8, 15]. The need for a novel parasite abundance screening tool is further emphasized by the data on anthelmintic resistance in sheep nematodes, which suggest prevalent issues regarding wide-spread treatment failures even in well-developed countries [4, 16]. However, the bioinformatics side of processing amplicon sequencing data obtained from NGS platforms is still an intimidating hurdle, not least because there are multiple different analysis tools and approaches available, each of varying difficulty and unknown impact on the final estimations of parasite abundance.

In this study, we focused on pragmatically describing and comparing three different pipelines (based on DADA2, Mothur and SCATA) and their impacts on the estimation of nematode species composition and richness in the most recently recovered paired Swedish sheep flock samples where treatments with anthelmintic substances were undertaken.

## Methods

### Samples

Samples were received from farms across south-central Sweden as part of a routine herd health monitoring program which is performed on commercial sheep farms experiencing recurrent problems with GIN infections. In total, 64 paired (before and after treatment; from 21 different farms) samples were collected between 2017 and 2020, of which 22 samples were taken before and after albendazole (ABZ) treatment (i.e. 11 before, 11 after), 34 were taken before and after ivermectin (IVM) treatment and eight were taken before and after monepantel (MOP) treatment (however MOP was only tested on 1 farm on different occasions). Samples were collected before and 10–14 days after treatment with the aforementioned anthelmintic drugs. Between 10 and 15 animals per flock were sampled on each occasion in the presence of a licensed veterinarian. The collected fecal samples were placed in marked zip-locked bags and sent via national post to the diagnostic laboratory where the FEC estimations were performed.

### FEC estimation and DNA extraction

Fecal egg counts for collected samples from sheep were determined by using a modified McMaster method as described previously [17]. The fecal samples were then used to hatch and harvest infective L3 as described previously [6]. DNA was extracted using the Nucleospin DNA tissue kit (Macherey–Nagel, Thermo Fisher Scientific, Waltham, MA, USA) according to the manufacturer’s guidelines.

### Library preparation and sequencing

Universal primers for nematode ITS2 ribosomal DNA (rDNA) amplification (NC1–NC2), tagged with an 8-bp barcode on the 5ʹ end of both NC1 and NC2, were used to generate ITS2 fragment libraries for each sample; these ITS2 fragment libraries were then pooled. Amplification, clean-up and control for concentration as well as purity of samples were carried out as described earlier [6]. After pooling the individual libraries at equal concentrations, sequencing was carried out on a PacBio SMRT cell V3 RSII system at SciLifeLab, Uppsala, Sweden.

### Bioinformatic analysis

The raw sequencing data (compressed*.fastq* files) for larvae pools were processed with three distinct pipelines involving DADA2 v.1.2 [18], SCATA (http://scata.mykopat.slu.se/) and Mothur v.1.46.1 [19] analysis software.

#### DADA2

DADA2 is a package for R, designed for inferring amplicon sequence variants (ASVs) from amplicon sequence data. The DADA2 approach for the analysis of ITS2 amplicon read data generated with the Illumina platform is readily available (https://www.nemabiome.ca/dada2_workflow.html) and was used here as a reference. Prior to this, the sequence data was demultiplexed using lima v.2.4 (*lima-hifi-prefix SYMMETRICS*; https://github.com/PacificBiosciences/barcoding). Demultiplexed reads were then subjected to primer removal using Cutadapt v.3.4 [20] in Rstudio (v.4.1.0, https://www.rstudio.com/) (system2 (cutadapt, args = c (R1.flags, R2.flags, ‘-n’, 2, ‘-o’, sample.cut [i], sample.[i]). Further filtering was employed to remove reads containing unresolved nucleotides (maxN = 0) as well as reads exceeding the expected error number (maxEE = 2) and size range (200–450 bp). The obtained dataset was then used as input in order to determine the error rates using the error function specifically adjusted for reads generated with the PacBio sequencing platform (learnErrors, *errorEstimationFunction* = *PacBioErrfun*). The same dataset was used to perform the removal of identical reads (derepFastq), while the composition of the sequenced pool was then inferred using the dereplicated sequence dataset as input (dada). Since PacBio sequencing data are unidirectional, read pairs did not need to be merged. Finally, chimeras were removed (removeBimeraDenovo; *method* = *‘consensus’*) and the taxonomic assignment of ASVs was carried out using *assignTaxonomy* together with the appropriate at the time (version 1.2) nematode ITS2 database as reference (https://www.nemabiome.ca/its2-database.html; in a regular FASTA format fit for analysis with DADA2) [21]. The missing taxonomic data was filled in by manually BLASTing the unresolved ASVs and selecting the most probable (based on *E*-value) species candidate.

#### Mothur

Mothur is a free, open-source software, adapted for multiple types of analyses of microbial communities and based on a custom command-line-type interface. The Mothur pipeline, as described on https://www.nemabiome.ca/mothur_workflow.html, was employed to analyze the previously demultiplexed (with lima v.2.4) and trimmed (with Cutadapt v.3.4) reads. For that purpose, demultiplexed reads for every barcode tag combination were concatenated and converted into a*.fasta* file (sed -n '1 ~ 4 s/^@/ > /p; 2 ~ 4p' in.fastq  > out.fasta), meanwhile the *.groups* file was obtained by extracting the headers of the said* .fasta* file (sed -n '1 ~ 2p' file  > file.out) and generating an additional column containing group (i.e. sample) names (sed -i "s/$/\t$group_name/). The contig generation step was skipped, and reads were filtered to retain sequences between 200 and 450 bp and not containing any ambiguous nucleotides (maxambig = 0). The remaining reads were aligned to the newest (at the time) available (v1.2) nematode ITS2 database (https://www.nemabiome.ca/its2-database.html; in the alignment format specifically for Mothur) [21], and only the sequences that were at least 90% similar to the entries in the database were kept. The minimum search score during the alignments was set to 10. The retained sequences were assigned to clusters using the k-nearest neighbor approach (*k* = 3).

#### SCATA

SCATA is a free, online web-based interface initially developed for the analysis of fungal communities, based on tagged ITS sequence amplicon data. In SCATA, raw amplicon sequences (in a* .fastq* format) and a tag-containing file (in a *.txt* format) are uploaded manually to the website without any prior processing. Sequence demultiplexing, clean-up and clustering with SCATA have been used previously to study metabarcoded ITS2 sequences belonging to GINs [6] and these steps were followed here to a major extent with a few exceptions, which we believed would improve the clustering of more rare species. Briefly, amplicon reads were removed if their mean nucleotide qualities were < 20 and if any of the individual nucleotide qualities was < 10. Read length was constrained between 200 and 450 bp. Demultiplexed and primer-free reads were assigned to operational taxonomic units (OTUs) using the clustering algorithm USEARCH [22] and at least a 85% overlap in the sequence length; a pairwise alignment was needed for the clustering of sequences into the same OTU. The threshold for clustering distance (i.e. dissimilarity) was set to 0.001, homopolymers longer than 3 bp were collapsed and rare genotypes appearing less than 10 times in the combined dataset were removed. The rest of the parameters were kept at their default values. Blast+ (https://www.ncbi.nlm.nih.gov/books/NBK279690/) along with the publicly available nematode ITS2 database (v.1.2) (https://www.nemabiome.ca/its2-database.html; in a regular FASTA format) [21] were utilized to assign the most probable nematode species to each OTU.

The obtained data tables, containing either ASVs or OTUs as well as taxonomic identifications for each, sample names and the number of pre-filtered reads (i.e. number of reads which were 200–450 bp and with no ambiguous nucleotides per sample per ASV/OTU) were subjected to further filtering. Due to the ambiguous nature of singleton reads, we chose to remove them as they can potentially inflate diversity [23, 24]. Two subsequent arbitrary hard cut-offs were set, as suggested previously [25], to remove other rare reads, which amounted to < 0.5% of the total read number for every sample and samples together, if their total minimum number of reads was  < 100. This step was an attempt to accurately measure species diversity and relative frequencies. Given that species diversity measures are also dependent on the number of sequences obtained from samples, random subsampling of all samples down to the read size of the smallest one could have reasonably been done [26]. On the other hand, random subsampling of reads to the smallest library size does not account for zero-inflated data and can possibly lead to the exclusion of valid data points and was thus not performed here [24]. The final dataset containing ASVs/OTUs, species names, sample names and the number of filtered reads had the reads for every sample merged if the ASVs/OTUs belonged to the same species.

For every approach, relative frequencies of each taxonomic unit (i.e. species) were calculated by dividing the species-specific reads by the total number of reads for every sample. Species richness for every sample in the respective category (either pre- or post-treatment) was estimated by summing the presence of each species. Alpha diversity, in the form of inverse Simpson (1/D) indices, was calculated for all samples processed with each pipeline (both pre- and post-treatment) using the *vegan* package (https://CRAN.R-project.org/package=vegan; v.2.6.2) for R. In order to determine the diversity indices, relative abundance data for species was adjusted by dividing each relative abundance value by the smallest in that dataset and rounding up to the closest integer.

Additional data manipulations (as well as illustrations) were made with (the *ggplot2* package v3.3.5 https://cran.r-project.org/web/packages/ggplot2/index.html for) Rstudio.

### Statistical analysis

One-way analysis of variance (ANOVA; using the package *stats* v.3.6.2 and function *lm* for R) was used to compare the relative frequencies of major nematode species, richness and alpha diversity (inverse Simpson indices) present in either pre-treatment or post-treatment samples as well as between the two groups, among the different pipelines.

## Results

### Sample description

A total of 114 ASVs (for 17 different species) were identified using the DADA2 pipeline, 218 sequence clusters (for 15 different species) were identified using the SCATA pipeline and analysis with Mothur yielded 13 different species. Following the dataset filtering steps, out of the initial 64 samples, 62 and 61 passed the set criteria for the DADA2 and Mothur pipelines, repectively, whereas 59 samples passed the set criteria for the SCATA pipeline. In total, 170,355 high-quality, filtered reads (an average of 2747.6 per sample [range 189–7140]) were obtained for the samples analyzed with the DADA2 pipeline. In comparison, the dataset obtained with Mothur resulted in 176,933 high-quality, filtered reads (an average of 2900.5 per sample [range 199–7417], and the analysis with SCATA yielded 68,037 high-quality, filtered reads (an average of 1153.1 per sample [range 110–2790]) (Additional file 1: Table S1).

**Fig. 1:**
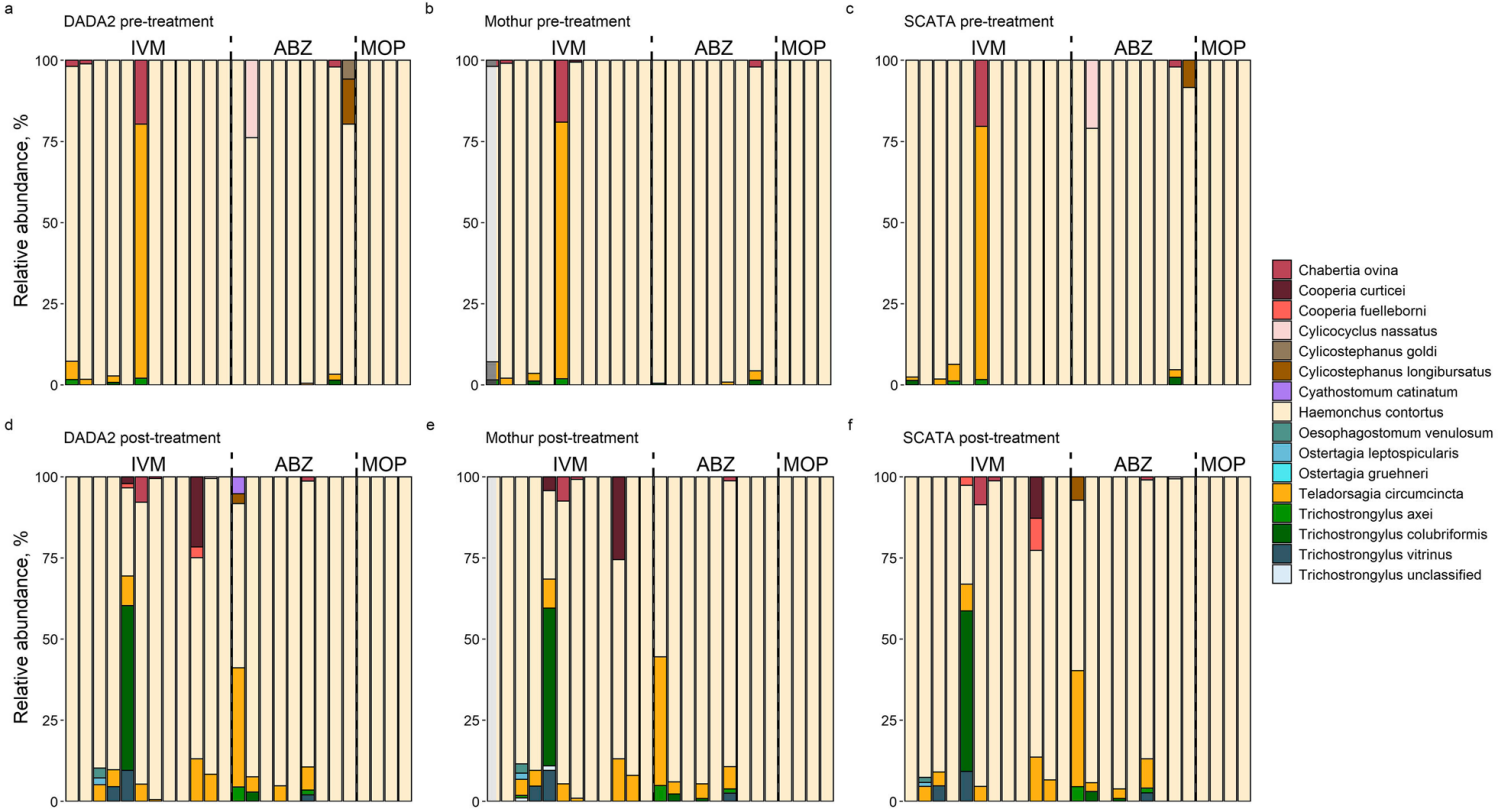
The species composition in analyzed, individual samples (25 pre- and 25 post-treatment) with three different pipelines.** a**–**c **Sample compositions in terms of fraction (%) of each individual species in pre-treatment samples with the DADA2 (**a**), Mothur (**b**) and SCATA (**c**) pipelines.** d**–**f** Sample compositions in terms of fraction (%) of each species in post-treatment samples with DADA2 (**d**), Mothur (**e**) and SCATA (**f**) pipelines. Each bar represents the composition of an individual sample, analyzed with a particular pipeline, and each color represents a different species, as indicated in the legend. Samples in both pre- or post-treatment categories were further subdivided into groups according to which drug was used to treat the flocks: ivermectin (IVM), albendazole (ABZ) or monepantel (MOP)

Out of the 62/61 samples for DADA2 and Mothur, 56 were kept (= 28 paired samples); for SCATA, 54 (= 27 paired samples) out of the initial 59 samples were kept. Due to missing samples (either before treatment, after treatment or both) for one or two of the pipelines, the final shared sample list, consisting of 50 samples (25 intact pairs; 9 paired samples for ABZ, 12 for IVM and 4 for MOP), was used for comparing species compositions, richness and diversity between the different bioinformatic pipelines. For all pipelines, *H. contortus* was found in 49 out of 50 retained samples, while the second most abundant species, *Teladorsagia circumcinta* (as well as all the others), was found to be more locally distributed, i.e. in 17 (with Mothur and DADA2) and 15 (with SCATA) of the 50 samples (Fig. 1).

### Analysis of the pre-treatment samples

**Fig. 2:**
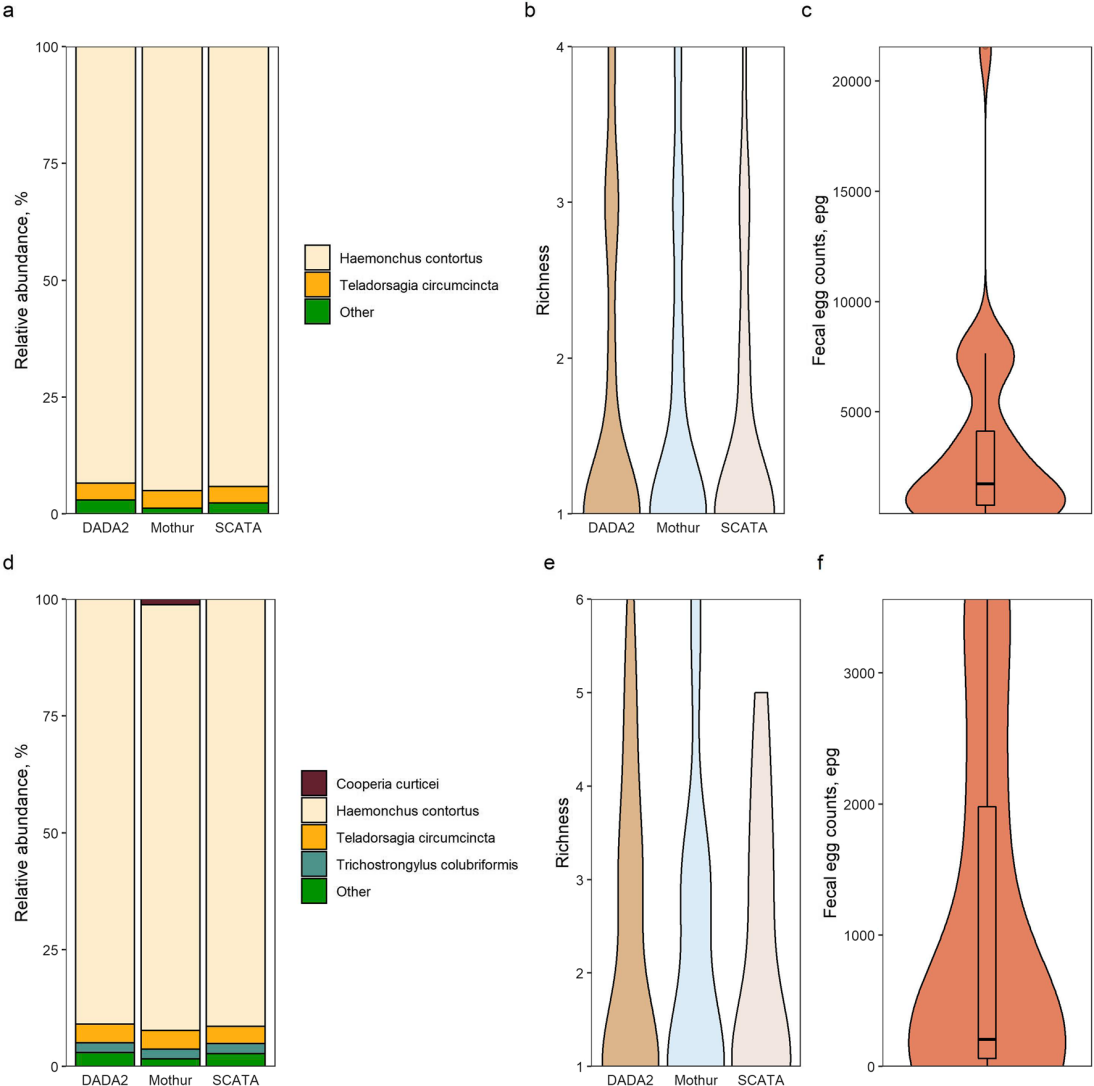
Comparisons of the mean species relative frequencies and species richness between the three pipelines both pre- and post-treatment, supplemented with egg count data. Mean fractions of each major (> 1%) species were compared between the pipelines for both pre- (**a**) and post-treatment (**d**) samples. Colors represent different species, or a collection of rare species, as indicated in the legend. Species richness was also compared between the pipelines for both pre- (**b**) and post-treatment (**e**) samples. Egg counts for samples pre-treatment (**c**) and post-treatment (**f**) are also shown. Box plots within the violin plots for egg counts represent the median values

The main species before treatment that was identified with all three pipelines were *H. contortus* (mean relative abundance 93–95%), followed by *T. circumcinta* (mean relative abundance 3.5–3.7%). Other, less abundant (≤ 1%) species consisted of *Chabertia ovina* (0.99%), *Cylicocyclus nassatus* (0.95%), *Cylicostephanus goldi* (0.23%), *Cylicostephanus longibursatus* (0.55%), *Trichostrongylus axei* (0.14%) and *Trichostrongylus colubriformis* (0.09%) with DADA2, *C. ovina (*0.95%*), Cooperia curticei* (0.02%), *T. axei* (0.14%) and *T. colubriformis* (0.12%) with Mothur and *C. ovina* (0.89%), *C. nassatus* (0.83%), *C. longibursatus* (0.33%), *T. axei* (0.11%) and *T. colubriformis* (0.14%) with the SCATA analysis pipeline (Fig. 2a–c; Additional file 2: Table S2). No statistically significant differences between the means of either *H. contortus* or *T. circumcinta* in the pre-treatment category were observed when comparing the different pipelines (one-way ANOVA, *df* = 74, *F* = 0.04, *P* = 0.96 and *df* = 74, *F* = 8e−04, *P* = 0.99, respectively). The species richness estimation likewise revealed no significant differences between the mean richness values obtained with all pipelines (one-way ANOVA, *df* = 74, *F* = 0.1, *P* = 0.83) (Fig. 2b). In agreement with previous data, alpha diversity indices for samples processed with the different pipelines showed no significant variation (one-way ANOVA, *df* = 74, *F* = 0.4, *P* = 0.62; Additional file 3: Figure. S1a). Mean FECs in the pre-treatment samples were 3536 ± 4601 (median value 1725) eggs per gram (epg) (Fig. 2c).

### Analysis of post-treatment samples

The major species in the post-treatment samples across all pipelines were *H. contortus* (mean relative abundance range: 90–91%), *T. circumcinta* (mean relative abundance range: 3.7–4.0%) and *T. colubriformis* (mean relative abundance range: 2.0–2.1%). With the Mothur pipeline *C. curticei* was found to be present at 1.2% relative abundance, while with the other two pipelines this species was found at  < 1% relative abundance (0.51% for SCATA and 0.94% for DADA2) and was therefore included in the *Other* species category. Rarer species present in the samples at  ≤ 1% relative abundance were also included in the *Other* category, among which were the following species: *C. ovina* (0.39%), *Cooperia fuelleborni* (0.18%), *Cyathostomum catinatum* (0.21%), *C. nassatus* (0.02%), *C. longibursatus* (0.12%), *Oesophagostomum venulosum* (0.12%), *Ostertagia leptospicularis* (0.08%), *T. axei* (0.23%) and *Trichostrongylus vitrinus* (0.64%) with DADA2; *C. ovina* (0.38%), *O. venulosum* (0.11%), *O. leptospicularis* (0.07%), *T. axei* (0.27%), *Trichostrongylus unclassified* (0.09%) and *T. vitrinus* (0.67%) with Mothur; and *C. ovina* (0.43%), *C. fuelleborni* (0.50%), *C. nassatus* (0.02%), *C. longibursatus* (0.29%), *O. venulosum* (0.06%), *Ostertagia gruehneri* (0.04%), *T. axei* (0.23%) and *T. vitrinus* (0.66%) with SCATA (Fig. 2d–f; Additional file 1: Table S1). No significant differences were observed when comparing the means of each major species with the three different pipelines post-treatment (one-way ANOVA, *df* = 74, *F* = 0.005, *P* = 0.99 for *H. contortus*; *df* = 74, *F* = 0.01, *P* = 0.98 for *T. circumcinta*; *df* = 74, *F* = 5e-04, *P* = 0.99 for *T. colubriformis*). No significant differences for either the mean values of species richness (one-way ANOVA, *df* = 74, *F* = 0.01 *P* = 0.98) or alpha diversity (one-way ANOVA, *df* = 74, *F* = 0.002, *P* = 0.99) post-treatment, obtained with the different pipelines, were found (Fig. 2e; Additional file 3: Figure S1b). Mean FECs in the post-treatment samples were 1055 ± 1341 (median value: 205) epg (Fig. 2f).

### Comparison between the treatment groups

Despite statistically significant changes in the observed epg counts post-treatment (one-way ANOVA, *df* = 49, *F* = 6.7, *P* = 0.01), comparing the mean relative frequencies of the three major species, i.e. *H. contortus*, *T. circumcinta* and *T. colubriformis*, before and after the subsequent treatment yielded no significant differences for all pipelines, which is unsurprising given the high relative abundance of likely resistant *H. contortus* DNA in the samples (one-way ANOVA, *df* = 49, *F* = 0.21, *P* = 0.63, *df* = 49, *F *= 0.55, *P* = 0.45 and *df* = 49, *F* = 0.26, *P* = 0.60 for *H. contortus* with DADA2, Mothur and SCATA respectively; *df* = 49, *F *= 0.01, *P* = 0.92, *df* = 49, *F* = 0.008, *P* = 0.92 and *df* = 49, *F* = 0.002, *P* = 0.96 for *T. circumcinta* with DADA2, Mothur and SCATA, respectively; and *df* = 49, *F* = 1, *P* = 0.31,* df* = 49, *F* = 0.99, *P* = 0.32 and *df* = 49, *F* = 1, *P* = 0.32 for *T. colubriformis* with DADA2, Mothur and SCATA, respectively). Nevertheless, on an individual level, samples such as IVM5 (change from 100% *H. contortus* pre-treatment to 49–51% *T. colubriformis* post-treatment), IVM6 (change from 79% *T. circumcinta* pre-treatment to 87% *H. contortus* post-treatment) and ABZ1 (change from 99–100% *H. contortus* pre-treatment to  50–55% *H. contortus* post-treatment) in Fig. 1 did show substantial changes in species compositions after treatment. Overall, no statistically significant changes in the means of species richness (one-way ANOVA, *df* = 49, *F* = 2.1, *P* = 0.14 [DADA2]; *df* = 49, *F* = 2.2, *P* = 0.14 [Mothur]; and *df* = 49, *F* = 3.7, *P* = 0.06 [SCATA]) or alpha diversity values (one-way ANOVA, *df* = 49, *F* = 2.2, *P* = 0.13 [DADA2]; *df* = 49, *F* = 3.6, *P* = 0.06 [Mothur]; and *df* = 49, *F* = 2.8, *P* = 0.1 [SCATA]) pre- and post-treatment across the three pipelines were observed.

## Discussion

Although several tools exist for the detection of GIN species as well as for the estimation of parasite community compositions, multiplexed library sequencing with a NGS platform has been shown to possess unique advantages in the form of higher sensitivity, specificity, increased throughput and limited bias [7, 8, 15]. In the present study, we applied this approach to study paired fecal samples from Swedish sheep before and after treatment with either IVM, ABZ or MOP. More importantly, motivated by the current gap in our knowledge regarding the methods to analyze data produced by NGS and the specific outcomes of choosing one bioinformatic pipeline over the other, we compared three different bioinformatic approaches to estimate the abundance of GIN species in 25 paired samples from Swedish sheep flocks.

### Outcomes of the pipeline comparisons

The flock samples studied here were recovered from multiple farms which have, according to the owners, struggled with persistent parasitism, likely due to anthelmintic resistance. Therefore, given the origin of these samples, it is unsurprising that the mean relative abundance of *H. contortus*, a species known to be capable of rapidly developing resistance to drugs [27, 28] and to be ubiquitous in Sweden [6], was so high (> 90% in both pre- and post-treatment categories). This finding seems to confirm that Swedish sheep farms experiencing recurring parasitic infections in flocks despite treatment tend to be disproportionately affected by *H. contortus*, while other less abundantly found species, such as *T. circumcinta* and even* Trichostrongylus* sp., appear to be more sporadically distributed across comparably fewer farms. In addition, among the rarest species (< 1% mean relative abundance) we identified several known horse strongyle parasites from the genera *Cylicocyclus*,* Cylicostephanus* and *Cyathostomum* as well as one so far only found in impalas and buffalo (*Cooperia fuelleborni*). It is unclear as to why these species were found within the collected fecal samples from sheep; however, the equine parasite species have also been found in sheep fecal samples at low levels in a previous study [6]. Given the relative rarity of these parasites in the recovered samples, it might be a simple case of sample cross-contamination during the initial phase(s) of sample recovery, storage and/or handling. At the same time, it cannot be excluded that the cross-contamination could have originated due to the fact that both sheep and horses sometimes co-graze on the same pastures. While these horse-infecting species were comparably rare—they were not picked up by the Mothur pipeline simply due to the fact that the current reference database (in the alignment format intended for use with Mothur and downloaded from https://www.nemabiome.ca/its2-database.html) does not contain any of the sequences for those species (i.e. sheep unspecific nematodes) unlike the database in a regular FASTA format used for both SCATA and DADA2 (despite them being obtained from the same source). While it is not uncommon that different analysis tools require input files of different formats, we hope that the reference alignment database for veterinary parasite ITS2 sequences, used specifically for Mothur, is updated in the future by the curators of the website.

The outcomes for the samples analyzed with the three pipelines yielded almost identical relative frequencies for the major species, namely *H. contortus*, *T. circumcinta* and *T. colubriformis*, in their respective treatment categories (Fig. 2a, d). In addition, it was shown that both species richness and diversity indices appeared to also be insignificantly affected by the chosen pipeline (Fig. 2b, e; Additional file 3: Figure S1), effectively demonstrating that all three pipelines, despite their differences, resulted in comparable species compositions, richness and diversity.

Despite the significant decrease in egg counts obtained post-treatment (Fig. 2c, f), mean relative frequencies of major species as well as richness and diversity measures remained insignificantly affected, which points to a rigid community structure wherein the dominant species is *H. contortus*, an observation consistent with the data obtained for samples collected in the previous study [6]. One noteworthy difference between the previous study and the work here is that instead of identifying *T. vitrinus* as one of the major species, we found *T. colubriformis* to be the dominant species of the genus *Trichostrongylus*. This illustrates the importance of performing multiple screenings in order to establish the precise abundance of different parasite species in a large geographical area.

### Influence of the NGS platform

Most of the recently published studies employing NGS to study the nemabiomes in various host animals have relied on the accurate evaluation of short reads produced by the Illumina sequencing platform [7, 13, 15, 29]. In fact, to our knowledge few studies exist on nematode parasites wherein the employed sequencing platform used has been PacBio [6]. This is perhaps not surprising given that the Illumina platform was introduced earlier as it is considered to be part of the second generation of DNA sequencing [30]. In addition, initial studies comparing the two platforms have shown that in most cases both the costs of sequencing using this platform and the error rates were lower [31]. Nevertheless, with the advent of HiFi read technology, PacBio has been capable of achieving  > 99.9% accuracy, while simultaneously accounting for the short-read bias inherent to the Illumina platform [32]. Furthermore, PacBio can and has been successfully adopted to study the species compositions of fungal communities [32, 33] and has also more recently been demonstrated to perform on par with the Illumina platform in terms of correctly classifying sequences belonging to different taxa from multiple organism kingdoms [34].

### Differences between the analysis software

The three pipelines compared here, i.e. DADA2, Mothur and SCATA, are fundamentally different in the way data is handled. Firstly, on the surface level, SCATA is a web-based, graphical user interface (GUI) tool which employs high-capacity computer clusters in order to process tagged, sequenced amplicon data and which requires little knowledge or experience in bioinformatics. In contrast, DADA2 and Mothur rely on the user inputting commands into a terminal-based interface, thus requiring previous computational knowledge. As a consequence, although SCATA offers the simplest and most straightforward way to process NGS data for a user with limited bioinformatic experience, it is also arguably the most cumbersome of the three pipelines to analyze nemabiome data, mostly because both Mothur and DADA2 can be run by employing pre-written scripts. In addition, having relatively little control over the data processing steps, in our case, resulted in poorer outcomes when comparing the numbers of high-quality, filtered reads obtained with the SCATA pipeline in comparison to the DADA2 or Mothur (68,037 for SCATA vs 170,355 with DADA2 and 176,933 with Mothur). This discrepancy was therefore the likely cause for fewer samples passing the downstream filtering with SCATA.

Both SCATA and Mothur employ hierarchical clustering, in the case of this study a single-linkage (i.e. nearest neighbor) approach to assign sequences to OTUs [25]. In order to classify sequences into clusters, typically, pairwise alignment algorithms (such as Needleman–Wunch) are preferred to global alignments because of the differences in ITS sequence length and variability. In the case of DADA2, Needleman–Wunch local pairwise alignments are used to classify sequences and subsequently global alignments between the sequences are used to identify chimeras and merge read pairs [18]. Nevertheless, several issues inherent to OTUs and their assignment exist. Mainly, de novo OTU assignments (such as performed with SCATA) are confined to the dataset they originate from, whereas closed-reference OTUs (performed with Mothur), generated by comparing sequences to the reference when assigning OTUs, do not capture the true variation outside of the reference sequence set used to assign them [35]. ASVs ameliorate these limitations, and a convincing argument could be made for the replacement of OTUs with ASVs. While a set of sequences and an occurrence table are produced for both OTUs and ASVs, software-generated ASVs (such as with DADA2) do not use arbitrary cut-off values for similarity between sequences [18, 36] but compose ASVs de novo based on 100% similarity between sequences and are thus capable of increased precision at a single nucleotide resolution. This is in major part because of the machine learning algorithms which are used to estimate error rates and correct reads, thereby eliminating increasing amounts of sequencing errors and spurious reads [18]. This effectively affords ASV-based methods not only higher resolution to discriminate the true biological diversity, but also makes ASVs reusable and reproducible in future datasets.

Nevertheless, although the ASV-producing algorithms are capable of estimating fine differences between sequences while better accounting for sequencing errors, a reasonable question to ask might be whether or not this technology is useful when compared to the more conventional OTU-generating methods, specifically in parasites of veterinary interest. Although some species, such as *H. contortus*, are known for their high genetic variability [37, 38], generally due to high fecundity, GINs are still multicellular eukaryotic organisms that possess complex gene structures with both intronic and exonic regions, with intronic regions more likely to accrue mutations [39]. High mutation rates and horizontal gene transfer in rapidly multiplying bacterial organisms, on the other hand, together could make ASV-producing methods more suited for bacteria rather than helminth parasites. In line with this reasoning and based on the results of the present study, we did not observe any significant differences between the major species abundance estimations between the three pipelines used. This essentially suggests that all three of these pipelines produce statistically indistinguishable data and are equally fit in estimating relative frequencies of helminth parasites, especially those surviving anthelmintic treatment.

Collectively, while analysis of reads produced by both paired-end Illumina and PacBio Hi-Fi are supported with all three pipelines, we recommend the more intuitive and easier SCATA approach to the less experienced users who want a single-step approach to demultiplexing and extracting amplicon read data through a GUI. Reads lost with this approach should not, based on the results here, lead to significant changes in the relative abundances of nematode species. However, it should be noted that this approach can be time-consuming, depending on the size of the supplied dataset and on the number of users using the platform at any given time. For those more experienced with either Linux, R software or similar software, DADA2 and/or Mothur might constitute a faster and more flexible choice for analyzing large datasets. If precise delineation between species is necessary, although not exactly useful for parasitic nematodes as shown in this study, DADA2 is a suitable tool. Nevertheless, we recommend that users experiment with different taxonomy classifiers apart from the default *assignTaxonomy* used here. The closed-reference OTU assignment approach used in Mothur could be considered intermediary in terms of difficulty and constitutes a straightforward approach to analyzing tagged amplicon data, however one should note that both an accompanying input*.group* file as well a reference sequence database in an ‘alignment’ format (briefly described here: https://mothur.org/wiki/alignment_database/) are required.

### Shortcomings of the present study and design

The main drawback of the present study is the relatively high fractional abundance of *H. contortus* in the recovered field samples; consequently, an argument could be conceived that a more diverse and variable (in terms of relative abundance) mock community would be preferred for the comparison of the analysis outcomes achieved with different bioinformatic pipelines. In addition, our current sampling approach was based on using a fixed amount of fecal matter for the hatching of larvae and the subsequent harvesting of the total L3 population. Although we posit that this approach is a robust and unbiased way of sub-sampling the total fecal matter collected from the flock, different sampling procedures based on harvesting a precise, consistent number of larvae (e.g. > 1000) or on only involving samples within a certain epg range, could be preferable.

It is important to acknowledge that both Mothur and DADA2 were initially tailored to process Illumina paired end reads, although the error estimation function, i.e. *errorEstimationFunction* = *PacBioErrfun*, in DADA2 can be successfully adapted to approximate error rates in PacBio sequencing reads. As a consequence, our approach was slightly modified in that the read merging step was skipped. Nevertheless, to our knowledge, there are no obvious constraints or explicit statements in the documentation of either of the two analysis softwares warning or preventing the user against utilizing the said software to analyze reads derived from the PacBio sequencing platform. Moreover, the data produced were statistically indistinguishable between the different approaches, suggesting that all three pipelines produce similar data no matter the chosen platform of analysis. We are also aware of correction factors which have successfully been applied to better approximate the true relative abundance of GIN species in sheep as determined through Illumina-generated reads [8]; however, we did not think those are applicable given the different NGS platform used here.

Although NGS of tagged ITS2 amplicons achieves a much greater throughput than the analysis of individual samples, together with increased precision and accuracy compared to more conventional relative species abundance estimation techniques, the technology is not without its limitations. The most apparent drawback is related to uneven locus amplification efficiency in different taxa with the same, universal primer pair (in this case NC1 and NC2) [40, 41]. Secondly, different numbers of rRNA gene clusters per genome in different species can also have a direct impact in estimating the relative abundance of said species in a sample [42].

## Conclusions

In the present study we utilized the most recently recovered, paired fecal samples (pre- and post-treatment with either ABZ, IVM or MOP) from Swedish sheep farms to investigate nematode community compositions with three distinct bioinformatic approaches (relying on DADA2, Mothur and SCATA) to evaluate the impact of the chosen bioinformatic pipeline on the sheep nemabiome estimation. We found an overwhelming presence and spread *of H. contortus* on sheep farms where resistance has previously been an issue (in 49/50 samples) and demonstrated that all three bioinformatic pipelines perform equally well, despite the many differences between them, when adapted to study sheep nemabiome sequencing data. We hope that the practical information and considerations provided in this paper benefit the reader in further pursuing individual amplicon sequencing as well as processing experiments.

## Supplementary Information

**Additional file 1: Table S1. **Sample names, anthelmintic drugs used on farms where the samples were collected, the time at which the samples were collected and the filtered, high-quality reads derived with the DADA2, Mothur or SCATA pipelines, respectively. Note: Sample names in red correspond to samples (*n*=50) chosen for the final analysis on the impact of the pipeline(s) on the estimation of species abundance, richness and diversity.

**Additional file 2: Table S2.** Mean relative frequencies of each nematode species in both pre- and post-treatment samples with the three analysis pipelines based on the DADA2, SCATA or Mothur pipelines, respectively.

**Additional file 3: Figure S1. **Alpha diversity (Inverse Simpson, 1/D) index comparisons between the different pipelines (DADA2, Mothur and SCATA) in both pre- (**a**) and post-treatment (**b**) sample categories.

## Data Availability

The ITS2 data used in this study is available upon request. The raw dataset has also been deposited to BioStudies (https://www.ebi.ac.uk/biostudies/) under the name S-BSST819.

## Abbreviations

ABZ: Albendazole
ASVs: Amplicon sequence variants
FEC: Fecal egg count
GIN: Gastrointestinal nematode
ITS2: Internal transcribed spacer 2
IVM: Ivermectin
MOP: Monepantel
NGS: Next-generation sequencing
OTUs: Operational taxonomic units
