# Supplementary material for: Estimation of the impact of three different bioinformatic pipelines on sheep nemabiome analysis
Source: Parasit Vectors. 2022 Aug 11;15:290. doi: 10.1186/s13071-022-05399-0 (PMC9373329; doi:10.1186/s13071-022-05399-0)
Supplement: Supplementary file 1 — Additional file 1: Table S1. Sample names, anthelmintic drugs used on farms where the samples were collected, the time at which the samples were collected and the filtered, high-quality reads derived with the DADA2, Mothur or SCATA pipelines, respectively. Note: Sample names in red correspond to samples (n=50) chosen for the final analysis on the impact of the pipeline(s) on the estimation of species abundance, richness and diversity. [file 13071_2022_5399_MOESM1_ESM.docx]

| **Samples** | **Anthelmintic** | **Time** | **Filt. reads DADA2** | **Filt. reads Mothur** | **Filt. reads SCATA** |
| --- | --- | --- | --- | --- | --- |
| X1 | MOP | post | 2892 | 3085 | 1118 |
| X2 | MOP | post | 2472 | 2588 | 961 |
| X3 | MOP | post | 2647 | 3109 | 1257 |
| X4 | MOP | post | 2756 | 3382 | 919 |
| X5 | IVM | pre | 4431 | 4633 | 1998 |
| X6 | IVM | post | 7140 | 7417 | 2595 |
| X7 | IVM | pre | 5877 | 7229 | 2595 |
| X8 | IVM | pre | 5564 | 5771 | 2470 |
| X9 | ABZ | pre | 6938 | 7306 | 2790 |
| X10 | IVM | post | 6221 | 6666 | 2554 |
| X11 | ABZ | post | 4321 | 4002 | 1917 |
| X12 | IVM | post | 5549 | 5760 | 2116 |
| X13 | ABZ | pre | 4564 | 3647 | 1905 |
| X14 | ABZ | post | 5420 | 6561 | 1771 |
| X15 | IVM | pre | 6088 | 6527 | 2426 |
| X16 | IVM | post | 5277 | 5723 | 2052 |
| X17 | IVM | pre | 259 | 258 | 110 |
| X18 | ABZ | pre | 1026 | 1085 | 345 |
| X19 | IVM | post | 215 | 219 | - |
| X20 | ABZ | pre | 189 | 199 | - |
| X21 | ABZ | post | 551 | 571 | 307 |
| X22 | IVM | post | 194 | 201 | - |
| X23 | IVM | post | 241 | 243 | - |
| X24 | ABZ | post | - | - | - |
| X25 | IVM | pre | 2667 | 2685 | 840 |
| X26 | IVM | post | 1960 | 2008 | 898 |
| X27 | ABZ | pre | 2524 | 2102 | 1023 |
| X28 | IVM | pre | 2752 | 2845 | 1217 |
| X29 | IVM | post | 2546 | 2569 | 518 |
| X30 | ABZ | post | 1616 | 1612 | 694 |
| X31 | ABZ | pre | 2668 | 2902 | 999 |
| X32 | IVM | pre | 1472 | 1479 | 555 |
| X33 | ABZ | post | 2042 | 2069 | 868 |
| X34 | IVM | pre | 1864 | 2016 | 871 |
| X35 | IVM | post | 2155 | 2201 | 948 |
| X36 | IVM | post | 2370 | 2491 | 1060 |
| X37 | ABZ | pre | - | - | 896 |
| X38 | IVM | pre | 1764 | 1774 | 628 |
| X39 | IVM | post | 1573 | 1587 | 579 |
| X40 | ABZ | post | 2066 | 2213 | 809 |
| X41 | IVM | pre | 2092 | 2190 | 778 |
| X42 | IVM | post | 2320 | 2350 | 625 |
| X43 | ABZ | pre | 1833 | 1844 | 786 |
| X44 | ABZ | post | 2027 | 2125 | 779 |
| X45 | ABZ | pre | 2402 | 2418 | 891 |
| X46 | IVM | pre | 2402 | 2418 | 891 |
| X47 | IVM | post | 2703 | 2765 | 1003 |
| X48 | ABZ | pre | 1856 | 2025 | 825 |
| X49 | ABZ | post | 2600 | 2723 | 903 |
| X50 | ABZ | post | 3391 | 3473 | 1027 |
| X51 | IVM | pre | 2513 | 2628 | 895 |
| X52 | IVM | post | 2487 | 2941 | 1206 |
| X53 | IVM | pre | 1907 | - | 520 |
| X54 | IVM | post | 2955 | 3114 | 1122 |
| X55 | IVM | pre | 3035 | 3220 | 1331 |
| X56 | IVM | post | 1575 | 1576 | 820 |
| X57 | IVM | pre | 1852 | 1893 | 719 |
| X58 | IVM | pre | 2131 | 2211 | 1096 |
| X59 | ABZ | pre | 2519 | 2713 | 1168 |
| X60 | ABZ | post | 2892 | 3029 | 1175 |
| X61 | MOP | pre | 2868 | 3014 | 1072 |
| X62 | MOP | pre | 2132 | 2317 | 887 |
| X63 | MOP | pre | 2380 | 2489 | 940 |
| X64 | MOP | pre | 2612 | 2722 | 969 |
