# Supplementary material for: Estimation of the impact of three different bioinformatic pipelines on sheep nemabiome analysis
Source: Parasit Vectors. 2022 Aug 11;15:290. doi: 10.1186/s13071-022-05399-0 (PMC9373329; doi:10.1186/s13071-022-05399-0)
Supplement: Supplementary file 2 — Additional file 2: Table S2. Mean relative frequencies of each nematode species in both pre- and post-treatment samples with the three analysis pipelines based on the DADA2, SCATA or Mothur pipelines, respectively. [file 13071_2022_5399_MOESM2_ESM.docx]

|  | DADA2 | | SCATA | | Mothur | |
| --- | --- | --- | --- | --- | --- | --- |
| Species | pre-treatment | post-treatment | pre-treatment | post-treatment | pre-treatment | post-treatment |
| *Haemonchus contortus* | 93.4 | 90.9 | 94 | 91.3 | 95 | 91 |
| *Teladorsagia circumcinta* | 3.6 | 4 | 3.5 | 3.7 | 3.7 | 4 |
| *Chabertia ovina* | 0.99 | 0.39 | 0.89 | 0.43 | 0.95 | 0.38 |
| *Cooperia curticei* | - | 0.94 | - | 0.51 | 0.02 | 1.2 |
| *Cylicocyclus nassatus* | 0.95 | 0.02 | 0.83 | 0.02 | - | - |
| *Cylicostephanus goldi* | 0.23 | - | - | - | - | - |
| *Cylicostephanus longibursatus* | 0.55 | 0.12 | 0.33 | 0.29 | - | - |
| *Cyathostomum catinatum* | - | 0.21 | - | - | - | - |
| *Cooperia fuelleborni* | - | 0.18 | - | 0.5 | - | - |
| *Oesophagostomum venulosum* | - | 0.12 | - | 0.06 | - | 0.11 |
| *Ostertagia leptospicularis* | - | 0.08 | - | - | - | 0.07 |
| *Ostertagia gruehneri* | - | - | - | 0.04 | - | - |
| *Trichostrongylus axei* | 0.14 | 0.23 | 0.11 | 0.23 | 0.14 | 0.27 |
| *Trichostrongylus colubriformis* | 0.09 | 2.1 | 0.14 | 2.1 | 0.12 | 2 |
| *Trichostrongylus vitrinus* | - | 0.64 | - | 0.66 | - | 0.67 |
| *Trichostrongylus unclassified* | - | - | - | - | - | 0.09 |
