# Supplementary figures and images for: Estimation of the impact of three different bioinformatic pipelines on sheep nemabiome analysis
Source: Parasit Vectors. 2022 Aug 11;15:290. doi: 10.1186/s13071-022-05399-0 (PMC9373329; doi:10.1186/s13071-022-05399-0)

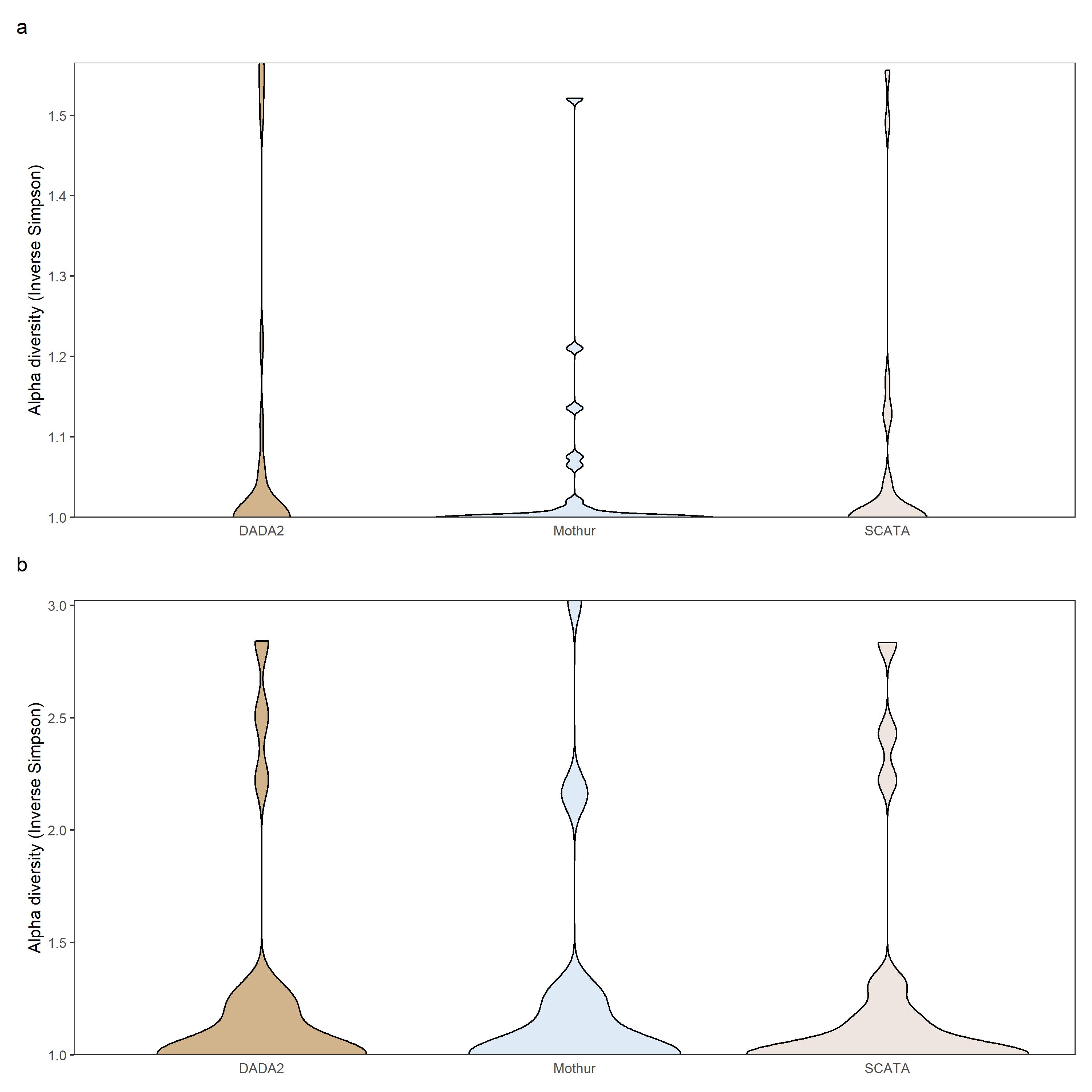

Supplement: Supplementary file 3 — Additional file 3: Figure S1. Alpha diversity (Inverse Simpson, 1/D) index comparisons between the different pipelines (DADA2, Mothur and SCATA) in both pre- (a) and post-treatment (b) sample categories. [file 13071_2022_5399_MOESM3_ESM.jpeg]
